# Speech-related parameters are sensitive measures of acetylcholinesterase inhibitor therapy in mild Alzheimer’s disease

**DOI:** 10.1371/journal.pone.0308409

**Published:** 2024-08-20

**Authors:** Éva Hegedűs, Magdolna Pákáski, Gábor Gosztolya, Ildikó Hoffmann, Ildikó Kovács, János Kálmán

**Affiliations:** 1 Department of Psychiatry, University of Szeged, Szeged, Hungary; 2 MTA-SZTE Research Group on Artificial Intelligence, Szeged, Hungary; Duke University Medical Center: Duke University Hospital, UNITED STATES OF AMERICA

## Abstract

Our aim was to find out whether speech-related temporal parameters (SRTPs) are sensitive indicators of the clinical outcome in acetylcholinesterase (AChE) inhibitor therapy with donepezil, compared to the standard cognitive Alzheimer’s Disease Assessment Scale-Cognitive Subscale (ADAS-Cog) used in clinical trials. In this 24-week-long, naturalistic, self-control, open-labeled, prospective pilot study with 10 mg donepezil on 20 mild AD patients, cognitive functions were evaluated using 15 different SRTPs analyzed by automatic speech recognition in the Speech-Gap Test® compared to ADAS-Cog test results. Among the SRTPs, the filled pause duration ratio significantly improved after 12 weeks of donepezil treatment. During the 24-week follow-up, additional SRTPs such as the filled pause count ratio and the filled pause frequency showed significant benefits. ADAS-Cog total scores showed a slight but not significant improvement compared to baseline after 12 and 24 weeks of donepezil treatment. Among the ADAS-Cog subtests, only orientation improved significantly after 24 weeks of donepezil treatment. Our results indicate that subtle changes in SRTPs measured by the Speech-Gap Test® could be considered as sensitive indicators of the efficacy of the pharmacotherapy in mild AD. According to our data, other cognitive domains did not show improvement in response to donepezil therapy rating by ADAS-Cog. Based on all of this, it is likely that examining and evaluating speech parameters may play an important role in determining the effects of pharmacological treatment of mild AD. The novelty of our study is that it applies the measurement of linguistic parameters as primary outcomes during a drug trial of mild AD in scientific research for the first time.

## Introduction

Continuous failures of drug development for Alzheimer’s disease (AD) have been experienced over the past two decades. The drugs used today to moderately slow the progression of AD, the acetylcholine esterase (AChE) inhibitors (AChEIs) and the NMDA antagonist memantine, were registered over 20 years ago. Since then, the US Food and Drug Administration (FDA) has not approved any drug that improves cognitive functions in AD. Last year, a retrospective evaluation of past clinical trials has revealed potential reasons for the failures in AD clinical trials [1]. According to this review, the poor choice of primary clinical outcome measures or insufficient testing for clinical efficacy might be causal factors. For more than 40 years to date, the Alzheimer’s Disease Assessment Scale Cognitive Subscale (ADAS-Cog) test has been the most commonly used primary clinical endpoint in failed clinical trials [2,3].

In recent years, numerous studies have achieved promising results in AD detection using automatic language processing [4]. The analysis of language functions can be a sensitive screening option in the identification of cognitive decline, an example of that is the Speech-Gap Test®, developed by our research team. In our earlier studies, applying the Speech-Gap Test®, mild cognitive impairment (MCI) and AD patients could be distinguished from healthy controls based on only temporal speech parameters [5–8]. Our more recent studies showed that the proposed properties of the Speech-Gap Test® and automatic speech analysis indeed carry clinically relevant information not only in Hungarian but also in English. The sensitivity of Speech-Gap Test was compared between English speaking and Hungarian speaking participants having mild cognitive impairment (MCI) or being healthy controls (HC). Seven temporal parameters in the English speaking sample and five in the Hungarian speaking sample differed significantly between the MCI and the HC groups. The English speaking sample showed 100% sensitivity on speech tempo and articulation tempo and high sensitivity (85.7%) on three more temporal parameters at moderate specificity. In the Hungarian speaking sample, high sensitivity (92.3%) was found on silent and total pause duration ratio, also on total pause average duration [9,10].

The first purpose of the present study is to determine whether the examination of language parameters could be capable of judging the beneficial effects of AChEI pharmacological treatment. Our further aim was to assess that examining the effects of AChEI on the speaking capacity of AD patients reaches or exceeds the sensitivity of the ADAS-Cog used in clinical trials to determine the severity of AD. Therefore, the efficacy of AChEI therapy was measured with the traditional outcome measures, such as the ADAS-Cog, and these results were compared to a novel, speech-based test method over 24 weeks. The aim of our study was to find and describe a new, rapidly feasible method that can be used to demonstrate the efficacy of drugs in AD not only in everyday medical practice, but also in the context of clinical trials.

## Methods

### Study design and participants

This was a naturalistic, self-control, open-labeled, single-center, prospective cohort study in the outpatient Memory Clinic of the Department of Psychiatry at the University of Szeged, Hungary. The research was performed according to the Declaration of Helsinki, with approval of Medical Research Council, Hungary (IV/2159-2 /2020/EKU). Written informed consents were signed by all subjects. The recruitment period for the study started on 24 June 2020 and ended on 19 November 2021.

Inclusion criteria were a minimum age of 60 years, a minimum of 8 years of formal education, and being Hungarian as the participants’ native language. All patients fulfilled the criteria outlined in the Fifth edition of the Diagnostic and Statistical Manual of Mental Disorders (DSM-5) and had probable AD according to the criteria of the National Institute of Neurological and Communicative Disorders and Strokes–Alzheimer’s Disease and Related Disorders Association (NINCDS–ADRDA) [11,12]. Exclusion criteria included major hearing problems (*e*.*g*. uncorrected hearing loss), manifest speech problems (*e*.*g*. any form of aphasia), significant articulation problems (*e*.*g*. stuttering), history of alcohol and substance use disorder, previous CT/MRI showing evidence of significant abnormality suggesting another potential etiology for dementia (*e*.*g*. vascular dementia or stroke), evidence of cerebral contusion, aneurysm, vascular malformations, and clinically significant space-occupying lesions, or the existence of depressive symptoms.

The cognitive assessment of AD patients was performed using a short neuropsychological test battery, including the Mini Mental State Examination (MMSE) [13], the Clock Drawing Test (CDT) [14] and the depressive symptoms were screened using the Geriatric Depression Scale (GDS) [15].

A total of 38 Caucasian subjects suffering from mild AD (60 years or older) were screened and included in the study (see Table 2). 18 individuals withdrew from the study due to adherence problems and due to somatic comorbidities. Therefore, 6 men and 14 women remained in the study.

The clinical diagnosis of AD was confirmed by initial evaluation through careful history taking (personal and family history), neurological and psychiatric examination by one of the authors, a neurologist (M.P.), along with computed tomographic scan (CT) or brain magnetic resonance imaging (MRI), also laboratory testing was performed to examine glucose level, B12, folate, complete blood count, liver function values, kidney and thyroid functions as well. Neuropsychological screening, monitoring, and language tests were conducted by one psychologist (É.H.), who was blind to the nature of the study.

### Procedure

After the clinical diagnosis of mild AD was established, the patients started AChEI therapy in the form of donepezil, according to the local dementia-related national healthcare professional guidelines [16]. The starting dose of 5 mg/day was increased to 10 mg/day by the 4th week. The first visit was before starting donepezil therapy, while the second and third visits were after 12 weeks and 24 weeks of donepezil therapy, respectively. During each visit, the Hungarian version of the ADAS-Cog [2,17] was administered and language samples were collected from each subject based on the protocol of the Speech-Gap Test® [10,18]. Speech-Gap Test® is time-efficient, it only requires 3–5 minutes to administer, compared to ADAS-Cog, in which case the mean time of administration is 30–40 minutes. Fifteen speech-related temporal parameters (SRTPs) were analyzed in the Speech-Gap Test® that are listed and defined in Table 1.

**Table 1:** Speech-related temporal parameters of spontaneous speech analyzed in the Speech-Gap Test®.

Parameters	Description
**Utterance lenght (s)**	The total lenght of speech (s)
**Articulation tempo (1/s)**	Total number of phones without hesitations (count) / total lenght of speech (s)
**Speech tempo (1/s)**	Total number of phones with hesitations (count) / total lenght of speech (s)
**Silent pause count ratio (%)**	Total number of silent pauses (count) x 100 / total number of phones (count)
**Filled pause count ratio (%)**	Total lenght of filled pauses (s) x 100 / total number of phones (count)
**Total pause count ratio (%)**	Total number of silent and filled pauses x100 / total number of phones (count)
**Silent pause duration ratio (%)**	Total lenght of silent pauses (s) x 100 / total lenght of speech
**Filled pause duration ratio (%)**	Total lenght of filled pauses (s) x 100 / total lenght of speech (s)
**Total pause duration ratio (%)**	Total lenght of silent and filled pauses (s) x 100 / total lenght of speech (s)
**Silent pause frequency (1/s)**	Total number of silent pauses (count) / total lenght of speech (s)
**Filled pause frequency (1/s)**	Total number of filled pauses (count) / total lenght of speech (s)
**Total pause frequency (1/s)**	Total number of silent and filled pauses (count) / total lenght of speech (s)
**Silent pause average lenght (s)**	Total lenght of silent pauses (s) /total number of silent pauses (count)
**Filled pause average lenght (s)**	Total length of filled pauses (count) / total number of filled pauses (count)
**Total pause average lenght (s)**	Total lenght of silent and filled pauses (s) / total number of silent and filled pauses (count)

#### Steps of Speech-Gap Test® administration procedure

Participants’ speech was recorded by an installed recorder application on mobile phone. Table 2 shows the details of spontaneous speech recording [10,18].

**Table 2:** Speech-Gap Test® procedure.

Steps	Description
**1.**	The investigator (1) shows the mobile phone device to the participant.
**2.**	The investigator (1) informs the subject that another colleague (Investigator 2) will call the mobile phone from another room.
**3.**	Investigator (1) informs the subject that the other Investigator (2) will provide the instuction for the task and they could not give any verbal prompts during the speech, they have to remain silent until the task is finished by the participant.
**4.**	Investigator (2) calls the mobile phone and after introducing themselves, asks the participant to talk about their previous day.
**5.**	Standardized instruction: ‘Please, tell me about your previous day in as much detail as you can.’
**6.**	After the participant finishes the task, Intvestigator (2) is allowed to speak again. He/she says good bye and finishes the phone call.

### Statistical analysis

Statistical analysis was performed using IBM SPSS (version 24) software. Descriptive statistics of demographic characteristics, of psychometric tests scores, and of temporal parameters of speech are expressed as means and standard deviations (SD). Normality test (Shapiro-Wilk tests) was applied for all variables. Based on the results, either the parametric repeated-measures ANOVA with Bonferroni Post-Hoc test or the non-parametric Friedmann-test with Kendall W coefficient were performed. The level of significance was set at *p<0*.*05*.

## Results

### Demographic parameters

Demographic characteristics and the baseline screening psychometric test results are presented in Table 3. According to the literary data, based on a frequency count of all individuals with AD, more women than men are living with a diagnosis of AD. The risk of developing AD for men is 6.3% and for women is almost twice of it, 12% [19]. Based on this, the 2:1 female-male ratio was applied in the study. The internal variation of the group for MMSE according to the Hungarian Dementia Guideline is mild dementia of selected patients as it ranges between 19 and 26 scores [16].

**Table 3:** Descriptive statistics of participants’ demographic characteristics and psychometric tests.

Demographic characteristics	Mean ± SD
**Sex (male/female)**	6 / 14
**Age (y)**	73.75 ± 5.5
**Education (y)**	12.15 ± 2.4
**Psychometric test scores**	
**MMSE**	22.10 ± 2.13
**CDT**	4.15 ± 3.28
**GDS**	3.40 ± 2.70

### Results of ADAS-Cog parameters

ADAS-Cog total scores slightly decreased compared to the baseline values after 12 and 24 weeks of donepezil treatment, but the difference was not significant (Table 4). As a result of 24 weeks of donepezil therapy, the ADAS-Cog total score showed no significant difference compared to the value of 12 weeks of donepezil therapy either (Table 4).

**Table 4:** Descriptive and comparative statistics of ADAS-Cog total and subtest scores; reference scores of non-demented patients [20] and mild AD patients (mAD) [21] and scores of the present study.

ADAS-Cog subtests	Reference scores (non-demented) (Mean ± SD)	Reference scores mAD (Mean ± SD)	Baseline scores (Mean ± SD)	Week 12 scores (Mean ± SD)	Week 24 scores (Mean ± SD)	F/ χ2	df	p
Word recall	3.19 ± 1.33	6.06 ± 2.04	5.93 ± 1.17	5.70 ± 1.18	5.71 ± 1.41	χ2 = 1.263	2	0.532
Commands	0.11 ± 0.34	1.14 ± 1.13	1.30 ± 0.92	1.10 ± 0.69	1.10 ± 0.83	0.772	2, 38	0.469
Constructional praxis	0.30 ± 0.49	0.66 ± 0.70	1.30 ± 1.13	0.75 ± 0.79	1.25 ± 0.97	2.673	2, 38	0.082
Naming objects and fingers	0.05 ± 0.22	1.08 ± 1.03	0.30 ± 0.57	0.25 ± 0.44	0.30 ± 0.57	0.192	2, 38	0.826
Ideational praxis	0.00 ± 0.00	0.42 ± 0.75	1.05 ± 1.15	1.30 ± 0.87	1.55 ± 1.32	χ2 = 3.569	2	0.168
**Orientation**	0.10 ± 0.31	2.16 ± 2.15	2.65 ± 1.35	2.05 ± 1.70	1.85 ± 1.76	**χ2 = 6.091**	2	**0.048**
Word recognition	1.17 ± 1.17	5.39 ± 3.59	6.40 ± 2.68	6.45 ± 2.84	5.25 ± 2.94	χ2 = 5.507	2	0.064
Remembering test instructions	0	1.39 ± 1.47	0	0	0.05 ± 0.22	1	2, 38	3.77
Spoken language	0.02 ± 0.15	0.67 ± 0.91	0	0	0	0	0	0
Word finding difficulties	0.01 ± 0.09	0.84 ± 0.98	0.05 ± 0.22	0	0	χ2 = 2	2	0.368
Comprehension of spoken language	0.03 ± 0.22	1.05 ± 0.99	0.90 ± 0.85	0.70 ± 0.66	0.70 ± 0.73	0	0	0
ADAS-Cog total score	4.98 ± 2.25	20.88 ± 10.07	19.89 ± 5.67	18.23 ± 5.06	17.68 ± 6.21	1.304	2, 18	0.296

**Fig 1:**
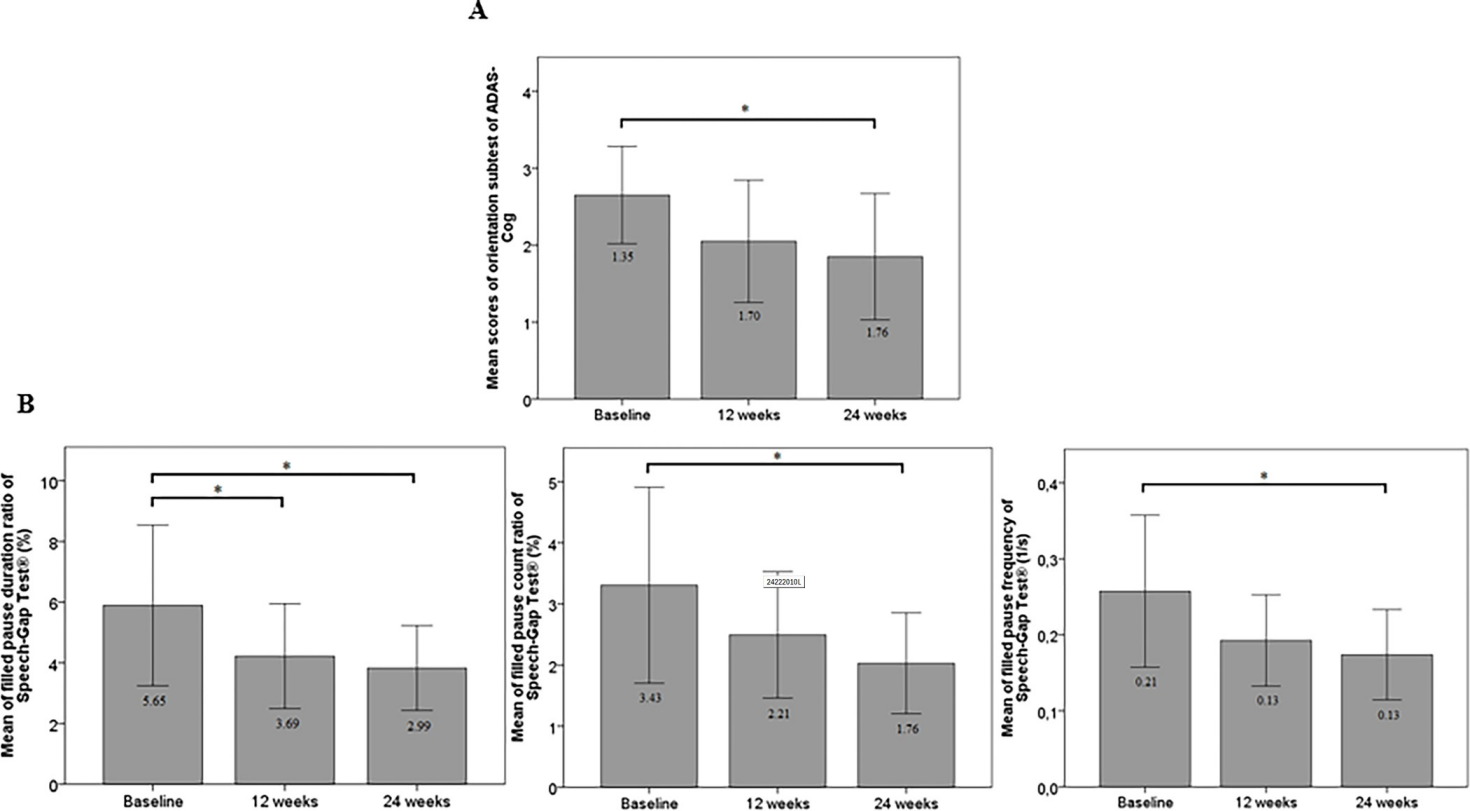
Cognitive changes due to 24th week donepezil therapy. A: Significant improvement in ADAS-Cog orientation task at 24th week of donepezil treatment, n = 20; *p = 0.*033*. B: Significant decrease in duration ratio of filled pause at 12th week and 24th week n = 20;*p = 0.*018*, furthermore in filled pause count ratio at 24th week n = 20;*p = 0.*002*, in filled pause frequency n = 20; *p = 0.*022* due to donepezil theraphy in mild Alzheimer’s disease patients. Data were analyzed by Friedmann-test and Kendall W coenfficient *<0.*05*.

Examining the ADAS-Cog subtests separately showed that 12 weeks of donepezil treatment did not induce improvement in any of the subtests. However, after 24 weeks of treatment, the value of orientation significantly decreased compared to the baseline (*p* = 0.033; Table 4, Fig 1A).

### Language-related outcome measures of the donepezil therapy

Before and during the donepezil treatment, the Speech-Gap Test® was also performed at all three visit occasions. From the 15 SRTPs tested, the filled pause duration ratio showed a significant improvement, as it decreased compared to the baseline values (*p* = 0.018) after applying AChEI drug for 12 weeks (Table 5, Fig 1B). At the 24-week follow-up, donepezil treatment induced a significant improvement in the values of 2 additional SRTPs beside the filled pause duration ratio. Significant differences were found between the baseline and the 24-week values of the filled pause count ratio (*p* = 0.002) and the filled pause frequency (*p* = 0.022), while the difference in the filled pause duration ratio showed further improvement (*p* = 0.018; Table 5, Fig 1B). The decrease in the filled pause duration ratio from the beginning of donepezil therapy during 24-week was continuous, but there was no significant difference between the 12-week and the 24-week values (Fig 1B).

**Table 5:** Descriptive and comparative statistics of speech related temporal parameters.

Speech related temporal parameters	Baseline (Mean ± SD)	Week 12 (Mean ± SD)	Week 24 (Mean ± SD)	F/ χ2	df	p
Utterance length (s)	63.43 ± 25.37	72.65 ± 32.03	76.07 ± 34.99	2.043	2, 18	0.159
Articulation tempo (1/s)	7.70 ± 2.08	8.10 ± 2.02	8.42 ± 1.81	3.228	2, 18	0.063
Speech tempo (1/s)	8.50 ± 2.03	8.85 ± 1.91	9.10 ± 1.78	2.276	2, 18	0.132
Silent pause count ratio (%)	6.76 ± 2.62	6.72 ± 2.60	5.79 ± 1.78	χ2 = 3.9	2	0.142
**Filled pause count ratio (%)**	3.31 ± 3.43	2.49 ± 2.21	2 .03 ± 1.76	**χ2 = 12.100**	2	**0.002**
Total pause count ratio (%)	10.17 ± 5.59	9.21 ± 4.47	7.82 ± 2.78	χ2 = 3.700	2	0.157
Silent pause duration ratio (%)	37.31 ± 14.55	37.46 ± 10.37	35.17 ± 11.53	1.280	2, 18	0.302
**Filled pause duration ratio (%)**	5.89 ± 5.65	4.22 ± 3.69	3.83 ± 2.99	**χ2 = 7.500**	2	**0.024**
Total pause duration ratio (%)	43.20 ± 15.31	41.68 ± 12.43	39.0 ± 11.94	2.962	2, 18	0.077
Silent pause frequency (1/s)	0.54 ± 0.16	0.56 ± 0.13	0.51 ± 0.13	χ2 = 2.100	2	0.350
**Filled pause frequency (1/s)**	0.26 ± 0.21	0.19 ± 0.13	0.17 ± 0.13	**χ2 = 7.300**	**2**	**0.026**
Total pause frequency (1/s)	0.80 ± 0.31	0.75 ± 0.21	0.68 ± 0.17	χ2 = 2.700	2	0.259
Silent pause average length (s)	0.77 ± 0.58	0.69 ± 0.20	0.72 ± 0.28	χ2 = 0.900	2	0.638
Filled pause average length (s)	0.22 ± 0.07	0.20 ± 0.06	0.21 ± 0.09	χ2 = 0.700	2	0.705
Total pause average length (s)	0.63 ± 0.51	0.56 ± 0.15	0.60 ± 0.23	χ2 = 0.900	2	0.638

## Limitations

Limitation of this study was the small sample size, which may had a negative impact on statistical power. By increasing the sample size may lead to more significant results, there is a probability that other temporal characteristics of speech could also mark the changes during donepezil therapy. Furthermore, the small sample size could be also a reason of only slight decrease in the ADAS-Cog total scores at the 12th and 24th week of donepezil treatment compared to the baseline values, these results might be improved by assessing a larger patient group.

## Discussion

The first main finding of the study in patients with mild AD is that donepezil treatment significantly improved speech tasks. This result proves that the evaluation of speech parameters could be more accurate in determining the effects of pharmacological treatments. The results of these mild AD patients conform to earlier review data indicating the specific effects of AChEI on the language parameters of patients with AD, especially during the moderate and severe stages of the illness [22]. Despite several SRTPs indicating significant improvements in the Speech-GAP Test®, no significant therapeutic response was detected neither by ADAS-Cog total scores and nor by most of the ADAS-Cog subtests during 12th or 24th weeks of donepezil treatment. This result shows that donepezil treatment improves only the speech domain in mild AD, but does not significantly improve other cognitive functions tested by the ADAS-Cog.

Donepezil (a reversible, non-competitive AChEI) was approved and became available for use in patients with mild to moderate AD in 1997 [23]. Since then, several randomized, double-blind, placebo-controlled clinical trials were conducted. In these studies, the efficacy of donepezil on cognitive functions was proven by the ADAS-Cog, which consists of 11 subscales designed to assess various cognitive abilities, including those associated with memory, language and praxis [24]. Several studies have shown that 7 of the 11 subtests of the ADAS-Cog, including naming of objects and of fingers, ideational praxis, commands, remembering test instruction, spoken language, word finding and comprehension of spoken language may be too easy for patients with milder AD [25–27]. Additionally, in the case of the 2 subtests examining language functions, spoken language and spoken language comprehension, the evaluation of performance depends on the subjective perception of the examiner and no literature data on their sensitivity is available. In addition, according to the present results, the ADAS-Cog subscores for language functions performed on mild AD patients in the 12th or 24th week of donepezil treatment did not indicate the effectiveness of the therapy.

The objective measurements of language functions provided by our artificial intelligence method showed a notable improvement in the reduction of the filled pause ratio SRTP, which can be interpreted as an indicator of the beneficial effects of donepezil treatment at both the 12-week and the 24-week follow-ups.

Furthermore, two additional SRTPs, the reductions in filled pause count ratio and the filled pause frequency also detected treatment response to donepezil at the 24-week assessment. Several former studies, both from our team and from other research groups, have previously shown that the presence of dementia or its prodromal stage, *i*.*e*. mild cognitive impairment, increased the presence and the duration of hesitation in spontaneous speech [6,8,10,28–34], and our previous study showed a significant increase in the number and in the duration of filled pauses among mild AD patients compared to healthy controls [8]. Our present results indicate that the decrease in the number and in the duration of filled pauses may be used to detect improvements in language functions elicited by donepezil treatment. Since the development of therapy is shifting towards interventions in the stage of AD that shows milder cognitive dysfunction, it is particularly important to prioritize reliable and quantitative assessment methods of language functions when evaluating drug therapy efficacy both in the context of clinical trials and during everyday patient care.

The application of sensitive methods is crucial in the monitoring of therapeutic efficacy, which can play an important role in new drug trials. Based on our study, SRTPs assessed by the Speech-Gap Test® can be a reliable measurement for the detection of therapeutic efficacy in patients with AD, especially when compared to currently standard neuropsychological tests, which did not detect significant changes in cognitive functions during the course of drug therapy. SRTPs indicated subtle changes, in contrast to the often used primary outcome measurement, the ADAS-Cog, which did not notice any difference in cognitive functions.

In conclusion, we provided evidence that temporal (acoustic) parameters of spontaneous speech are able to detect subtle changes during donepezil therapy in mild AD patients. According to the data, other cognitive domains did not show improvement in response to donepezil therapy ratings by the ADAS-Cog. Based on this, it is likely that examining and evaluating speech parameters may play an important role in determining the effects of pharmacological treatment of mild AD.

Our results suggest that the attributes of filled pause count ratio, filled pause duration ratio and filled pause frequency may be reliable indicators of cognitive function improvements during AChEI medication. Analysis of the SRTPs by the Speech-Gap Test® is a non-expensive, non-invasive, time-efficient, easy to use, objective and automatic tool to assess language functions. In addition, the process of recording spontaneous speech is less stressful for the patients compared to the performing of neuropsychological tests. Furthermore, the novelty of our study is that it applies the measurement of speech parameters as primary outcomes in a clinical trial of AD for the first time.

Language analysis has a great potential to become a primary outcome measure in future clinical trials in AD by providing sensitive and accurate measurements of cognitive functions through the evaluation of spontaneous speech, possibly increasing the chances of identifying effective compounds.
